# Knowledge, attitude, and practice towards COVID-19 among chronic disease patients at Aksum Hospital, Northern Ethiopia, 2020: a cross-sectional study

**DOI:** 10.1186/s40733-021-00074-0

**Published:** 2021-06-21

**Authors:** Assefa Iyasu, Berihu Hailu Kidanu, Kidane Zereabruk

**Affiliations:** School of Nursing, Aksum University, PO. box 298, Aksum City, Tigray Ethiopia

**Keywords:** COVID-19, Knowledge, Attitude, Practice, Chronic disease

## Abstract

**Background:**

The Coronavirus disease 2019 outbreak is the first reported case in Wuhan, China in December 2019 and suddenly became a major global health concern. Currently, there is no vaccine and treatment have been reported. The aim of this study was to assess the knowledge, attitude, and practice of COVID-19 among chronic disease patients.

**Methods:**

A hospital-based cross-sectional study was conducted among 422 chronic disease patients from July 01 to August 30, 2020 at Aksum Hospital, Northern Ethiopia. Both bivariable and multivariable logistic regression analyses with 95% confidence intervals were fitted to identify factors associated with poor knowledge and practice towards COVID-19. The adjusted odds ratio (AOR) was used to determine the prevalence of the association between the dependent and independent variables. A *P*-value < 0.05 was identified as statistically significant.

**Results:**

A total of 422 participants participated in this study, with a 100% response rate. The prevalence of poor knowledge, poor practice and unfavorable attitude was 35.1, 48.8, and 40.5%, respectively. Age (AOR = 1.5, 95% CI: (1.411, 2.432)), educational status of “can’t read and write” (AOR = 1.4, 95% CI: (1.332, 9.612)), and rural residence (AOR = 3.12, 95% CI: (2.568, 11.532)) were significantly associated with poor knowledge. Educational status of “can’t read and write” (AOR = 2.7, 95% CI (1.03–7.29)), and rural residence (AOR = 2.7, 95% CI (1.09–6.70)) were significantly associated with poor practice.

**Conclusions:**

The prevalence of poor knowledge and poor practice among chronic disease patients were high. Rural residence and educational status with “can not read and write” were significantly associated with poor knowledge and poor practice. Older age was significantly associated with poor knowledge.

## Introduction

An outbreak of pneumonia caused by a novel coronavirus occurred in Wuhan, Hubei province, in December 2019 and has spread rapidly throughout China [1].

On January 30, 2020, the World Health Organization (WHO) declared the outbreak a Public Health Emergency of International Concern and on February 12, 2020, the WHO named the disease caused by the novel coronavirus “coronavirus disease 2019” (COVID-19) [2].

The current COVID-19 pneumonia pandemic, caused by the severe acute respiratory syndrome coronavirus 2 (SARS-CoV-2) is spreading globally at an accelerated rate [3].

Six months after the outbreak of COVID-19 in Wuhan City, China, and about three months after the WHO declared it a pandemic, the disease had infected nearly 8 million people and killed more than 433 thousand globally [4].

Because of the high volume of air traffic and trade between China and Africa, Africa is at a high risk for the introduction and spread of COVID-19 [5]; although only Egypt has reported the first case on Feb 14, 2020 [6].

By June 30, 2020, approximately 16.3 million people in Africa will contract COVID-19 [7]. On 13 March, 2020, Ethiopia confirmed its first case of COVID-19 and on 26 June, 2020, tested 237,464 suspects, of whom 5425 (2.3%) cases had been confirmed positive and Outof the confirmed positive about 89 (1.6%) died and 1688 (31.1%) recovered [8].

Most of the fatal cases and severe illnesses like acute respiratory distress syndrome (ARDS) occur in aged and people who have underlying medical co-morbidities like diabetes, cancer, hypertension, heart, lung, and kidney diseases [9]. The spread of COVID-19 is still alarmingly increasing from day to day and is not controlled, even though there is health promotion, including hand washing and social distancing. Poor understanding of the disease among the community enhances the spread of infection and death. Therefore, successful prevention and minimization of morbidity and mortality due to COVID-19 require changing the behavior, which is influenced by individuals knowledge and attitude [10].

Therefore, understanding the “high-risk” groups, especially those with chronic disease, knowledge, attitude and practice (KAP) and possible risk factors is necessary and helps to anticipate the outcomes of planned behavior on COVID-19. Thus, this study aimed to determine the KAP towards COVID-19 and associated factors of poor knowledge and practice among chronic disease patients at Aksum Hospital. The results of this study in the early stages of the pandemic may help to direct the efforts and plans of public health authorities, clinicians, and the media of the country for better and timely control of COVID-19.

## Methods

### Study setting and period

A hospital-based cross-sectional study was conducted from July 01 to August 30, 2020, at Aksum Hospital in Aksum Town, Northern Ethiopia.

### Source and study population

All patients with chronic diseases (hypertension, diabetes mellitus, heart disease, chronic lung disease, and other diseases) who attended the chronic disease follow-up clinics at Aksum Hospital were the sources of population, while all patients with chronic diseases who attended the chronic disease follow-up clinics at Aksum Hospital during the study period were the study population.

### Inclusion and exclusion criteria

All chronic disease patients whose aged greater than 18 years who were on follow-up at the hospital during the study period were included whereas critically ill or had cognitive impairment and health professionals were excluded from this study.

### Sample size and sampling procedure

The sample size was calculated using a single proportion formula, n= $\frac{{\left[\mathrm{Z}\ \frac{\alpha }{2}\right]}^2\ \mathrm{p}\left(1-\mathrm{p}\right)}{d^2}$ at 95% confidence level, where Z $\frac{\alpha }{2}$ = standard normal deviation (1.96), d = 5% of marginal error, and p = prevalence 50%. Since, there are no published data that show the knowledge, attitude, and practice of COVID-19 among chronic disease patients in Ethiopia.

$$ \mathrm{n}=\frac{\ (1.96)2\times 0.5\ \left(1-0.5\right)}{(0.05)^2}=384 $$

There of 10% of the sample size would add for the non-response rate so that it would be a 422 study subjects. A consecutive sampling method was employed to select the study participants.

### Data collection tools and procedures

Prior to data collection pre-test was conducted on 10% of the study subjects which were 42 patients in Shire Hospital two weeks before the actual data collection period to ensure quality, clarity, understand and completeness of the data. The data were collected using a pre-tested, structured interviewer administered questionnaire. The questionnaire included socio-demographic and clinical characteristics and KAP towards COVID-19. Four BSc holder nurses were recruited for data collection and one MSc holder nurse was recruited as a supervisor by wearing a mask and glove in a well-ventilated room with a minimum distance of 2 m from the patients.

### Operational definitions

#### Knowledge towards COVID–19

The questionnaire regarding knowledge was sixteen questions (with minimum score 0 and maximum score 16); were answered on a yes/no basis and an additional “I don’t know” option. A correct answer was given 1 point and an incorrect/unknown answer was given 0 points. The total knowledge score ranged from 0 to 16. Participants’ overall knowledge was categorized using Bloom’s cutoff point, as good if the score was between 80 and 100% (12.7–16 points), moderate if the score was between 60 and 79%(9.6–12.6 points), and poor if the score was less than 60% (< 9.6 points) [11].

#### Practice towards COVID–19

Similarly, the question regarding the practice was fifteen (with a minimum score of 0 and a maximum score of 15); were answered yes or no, the correct answer was given 1 point and an incorrect answer was given 0 points. The overall practice score was categorized using Bloom’s cutoff point, as good if the score was between 80 and 100% (12–15 points), moderate if the score was between 60 and 79% (9–11.9 points), and poor if the score was less than 60% (< 9 points) [11].

#### Attitude towards COVID–19

The question regarding attitude was eleven (with a minimum score of 11 and a maximum score of 55). The score of the attitude was based on 5 points Likert scale, in which a score of 1 to 5 was given from strongly disagree to strongly agree. A mean score > 33 (answering strongly agree or agree) was carried out as a favorable e attitude and a score of 11 to 33 indicated an unfavorable attitude (answering strongly disagree or disagree or neutral) [12, 13].

### Data quality assurance

To assure the quality of data, a pretest was done on 10% of the subjects in Shire hospital having similar socio-demographic characteristics two weeks before the actual data collection. The reliability of the knowledge, attitude, and practice questionnaires were checked and the values of Cronbach’s alpha were 0.855, 0.793, and 0.795, respectively, indicating acceptable internal consistency [14]. Four BSC nurses and two supervisors were recruited for data collection and training was given. The collected data were checked by a supervisor and principal investigator daily.

### Data processing and analysis

After data collection, the data were entered in to Epidata 3.1 and exported to the SPSS version 22 statistical package for analysis. Descriptive characteristics are presented in the text, frequency percentage tables, and graphs. A binary logistic regression model was used to identify factors associated with poor knowledge and poor practice. Then, variables that were found to have an association with the outcome variable at *P* < 0.25 was used in multivariable logistic regression. Finally, a *P*-value < 0.05 was describe as a statistically associated with a 95% confidence level.

## Results

### Socio-demographic and clinical characteristics

Overall, 422 participants were included in this study, with a response rate of 422 (100%). Of the total participants, 220(52.1%) were female and the remaining were male. The mean age of the participants was (32.99 ±( 6.325) SD years) and whose age of 50–92 participants was 185(43.8%). Of the participants, 416(98.6%) and 6(1.4%) were Tigrian and Amhara, respectively. Of the total participants, 268(63.5%) were married. Regarding educational status, 182(43.1%) of the study participants could not read and write, while 133 (31.5%) could read and write. Of the total participants, 332(78.7%) were from an urban area. Of the study participants, 104 (24.6%), 95 (22.5%), and 90 (21.3%) were diabetes, hypertension and chronic lung disease, respectively (Table 1).

**Table 1:** Socio-demographic and Clinical Characteristics of Chronic Disease Patients, Aksum Hospital, Northern Ethiopia, 2020 (*N* = 422)

Variable	Category	Frequency N (%)
**Age**	18–34 years	73(17.3)
	35–49 years	164(38.9)
	50–92 years	185(43.8)
**Sex**	Male	202(47.9)
	Female	220(52.1)
**Residence**	Urban	332(78.7)
	Rural	90(21.3)
**Marital status**	Single	21(5)
	Married	268(63.5)
	Divorced	44(10.4)
	Widowed	89(21.1)
**Ethnicity**	Amhara	6(1.4)
	Tigray	416(98.6)
**Educational status**	Un able to write read	182(43.1)
	Able to write and read	133(31.5)
	Primary school	47(11.3)
	Secondary and above	60(14.2)
**Occupation**	House wife	78(18.5)
	Private business	90(21.3)
	Government employee	54(12.8)
	Farmer	95(22.5)
	Unemployed	62(14.7)
	Daily labourer	10(2.4)
	Retired	33(7.8)
**Type of chronic Disease**	Diabetes mellitus	104(24.6)
	Hypertension	95(22.5)
	Heart Disease	110(26.1)
	Chronic lung disease	90(21.3)
	Other^a^	23(5.4)
**High-risk group for developing severe illness**	Old age(elderly)	200(47.4)
	DM or HTN or Heart disease co-morbidity	150(35.5)
	Suppressed immunity	40(9.5)
	Chronic lung diseases	20(4.7)
	Children	5(1.2)
	Pregnant	7(1.7)

Notes: ^a^HIV; Rheumatoid arthritis, age category [15]

### Knowledge towards COVID–19

The prevalence of poor knowledge was 35.1% (95% CI (27.6–39.3%). About 102 (24.2%) study participants reported that COVID-19 symptoms appeared within 2–14 days. “Persons with COVID-19 cannot infect the virus to others if they have no any symptoms of COVID-19”, “washing hands frequently with soap and water for at least 20 seconds or use an alcohol-based hand sanitizer (60%) is important to prevent infection with COVID-19”, “isolation and treatment of people who are infected with the COVID-19 virus are effective ways to reduce the spread of the virus” by 336(55.9%), 320(75.8%), and 330(78.2%) of the study participants, respectively. People who have contact with someone infected with the COVID-19 virus should be immediately isolated in a proper place by 258(61.1%) participants. Most 310(73.5%) of the study participants reported that wearing masks when moving out of home is important to prevent infection with COVID-19 virus. Most 350 (82.9%) of the study participants reported that avoiding going to crowded places such as public transportations, religious places, hospitals and workplaces to prevent COVID-19 infection (Table 2).

**Table 2:** Knowledge of chronic disease patients, Aksum Hospital, Northern Ethiopia, 2020 (*N* = 422)

Serial Number	Knowledge Questions	Frequency (%)		
		Yes N (%)	No N (%)	I don’t know N (%)
1.	Main clinical symptoms of COVID-19 are fever, cough, shortness of breath, and fatigue	**389 (92.2)**	11 (2.6)	22 (5.2)
2.	Unlike the common cold, stuffy nose, runny nose, and sneezing are less common in persons infected with the COVID-19 virus	**150 (35.6)**	206 (48.8)	66 (15.6)
3.	COVID-19 symptoms appear within 2–14 days	**102 (24.2)**	24(5.7)	297 (70.1)
4.	Currently, there is no effective treatment or vaccine for COVID-19, but early symptomatic and supportive treatment can help most patients to recover from the infection	**300(71.1)**	3 (0.7)	119 (28.2)
5.	Not all persons with COVID-19 will develop severe cases. Those who are elderly, have chronic illnesses, and with suppressed immunity are more likely to be severe cases	**302 (71.5)**	18 (4.3)	102(24.2)
6.	Touching or shaking hands of an infected person would result in the infection by the COVID-19 virus	**286 (67.8)**	42 (9.9)	94 (22.3)
7.	Touching an object or surface with the virus on it, then touching your mouth, nose, or eyes with the unwashed hand would result in the infection by the COVID-19 virus	**222 (52.6)**	85 (20.1)	115 (27.3)
8.	The COVID-19 virus spreads via respiratory droplets of infected individuals through the air during sneezing or coughing of infected patients	**357 (84.6)**	13(3.1)	52(12.3)
9.	Persons with COVID-19 cannot infect the virus to others if he has no any symptom of COVID-19	236 (55.9)	**86 (20.4)**	100(23.7)
10.	Wearing masks when moving out of home is important to prevent the infection with COVID-19 virus	**310 (73.5)**	27 (6.4)	85(20.1)
11.	Children and young adults do not need to take measures to prevent the infection by the COVID-19 virus	130(30.8)	**200(47.4)**	92(21.8)
12.	To prevent the COVID-19 infection, individuals should avoid going to crowded places such as public transportations, religious places, Hospitals and Workplaces	**350(82.9)**	12(2.8)	60(14.2)
13.	Washing hands frequently with soap and water for at least 20 s or use an alcohol based hand sanitizer (60%) is important to prevent infection with COVD-19	**320(75.8)**	40(9.5)	62(14.7)
14.	Traveling to an infectious area or having contact with someone traveled to an area where the infection present is a risk for developing an infection	**400(94.8)**	1(0.24)	21(5)
15.	Isolation and treatment of people who are infected with the COVID-19 virus are effective ways to reduce the spread of the virus	**330(78.2)**	17(4)	75(17.8)
16.	People who have contact with someone infected with the COVID-19 virus should be immediately isolated in a proper place.	**258(61.1)**	32(7.6)	132(31.3)

Note: Numerical values printed in bold indicate the frequency of proper responses

### Associated factors of poor knowledge towards COVID-19

Crude associations of socio-demographic variables with poor knowledge were checked and age, educational status, marital status, residence, and occupation were statistically significant at *p* < 0.25. These variables having significant crude associations were entered into the multivariable logistic regression model. Accordingly, age, rural residence, educational status of “can’t read and write” were significantly associated with poor knowledge at *p* < 0.05.

This study showed that whose age of a 50–90 years old was 1.5 times more likely to be associated with poor knowledge than those aged a 18–34 years (AOR = 1.5, 95% CI: (1.411, 2.432)). This study also showed that participants who “can’t read and write” had 8.5 times more likely associated to poor knowledge than with an educational status of “secondary and above” (AOR = 8.52, 95% CI: (5.241, 23.543)), and the odds of having poor knowledge in rural residents were seven times (AOR = 7.21, 95% CI: (6.763, 13.392)) (Table 3).

**Table 3:** Factors associated with poor knowledge among chronic disease patients in bivariable and multivariable logistic regression analyses, Aksum Hospital, Northern Ethiopia, 2020 (*N* = 422)

Variable	Category	Poor knowledge		OR (95% CI)		
		Yes (***n*** = 148)	No (***n*** = 274)	COR	*P*-value	AOR
**Age**	18–34 years	10(13.7)	63(86.3)	1		1
	35–49 years	37(22.6)	127(77.4)	0.544(0.451, 2.097)	0.056	1.922(0.937, 3.939)
	50–92 years	101(54.6)	84(45.4)	0.132(0.101,1.209)*	**0.03**	1.5(1.411, 2.432)**
**Sex**	Male	81(40.1)	121(59.9)	1		
	Female	67(30.5)	153(69.5)	1.52(0.821,1.91)		
**Ethnicity**	Amhara	2(33.3)	4(66.7)	1		
	Tigray	146(35.1)	270(64.9)	0.924(0.821, 2.652)		
**Residence**	Urban	66(19.9)	266(80.1)	1		1
	Rural	82(92.2)	8(8.8)	0.024(0.017,6.051)*	**0.027**	7.21(6.763,13.392)**
**Educational level**	Un able to write and read	110(60.4)	72(39.6)	13.75(11.453, 57.893)*	**0.006**	8.52(5.241,23.543)**
	Able to write and read	20(15)	113(85)	1.59(1.001,8.213)*	0.07	1.89(0.978,6.519)
	Primary school	12(25.5)	35(74.5)	3.08(0.941,9.196)		0.71(0.673,4.921)
	Secondary and above	6(10)	54(90)	1		1
**Occupational status**	House wife	32(40.1)	46(58.9)	1		
	Private business	14(15.6)	76(84.4)	3.77(2.064,4.751)		
	Government employee	15(27.8)	39(72.2)	1.81(0.111,3.739)		
	Farmer	45(47.4)	50(52.6)	0.77(0.482,8.235)		
	Unemployed	27(43.5)	35(56.5)	0.9(0.261,5.94)		
	Daily laborer	8(80)	2(20)	0.17(0.085,4.743)		
	Retired	7(21.2)	26(78.8)	2.6(1.302,6.629)		

Notes: *p-value < 0.25, statistically significant in COR; ** *p*-value < 0.05, statistically significant in AOR. *Abbreviations*: *AOR* adjusted odds ratio, *CI* confidence interval, *COR* crude odds ratio. Age category [15]

### Attitude of chronic disease patients toward COVID–19

The prevalence of unfavorable attitudes was 40.5% (95% CI (32.1–46.4%). Most 302(71.6%) of the study participants strongly disagree with the statement “Ethiopia can win the battle against the COVID-19 virus”. Two hundred forty five (58.1%) had agree with “worried one of your family members may get an infection”. Among the study participants, “Prevalence of COVID-19 can be reduced by the active participant of health care workers in hospital infection control programs” 155(36.7%), “if a COVID-19 vaccine was available, I would have it” 316(74.9%), and “Patients should disclose their exposure” 381(90.3%) agreed, strongly agree, and strongly disagree, respectively (Table 4).

**Table 4:** Attitude of chronic disease patients, Aksum hospital, Northern Ethiopia, 2020 (*N* = 422)

Serial Number	Questions
1.	Do you agree that COVID-19 will finally be successfully controlled?
2.	Do you have confidence that Ethiopia can win the battle against the COVID-19 virus?
3.	You think you will probably get the illness
4.	You are worried one of your family members may get an infection
5.	If getting COVID-19, you will accept isolation in health facilities
6.	Transmission of COVID-19 can be prevented by washing hands with soap frequently
7.	Prevalence of COVID-19 can be reduced by the active participant of health care workers in hospital infection control programs
8.	If a COVID-19 vaccine was available, I would have it
9.	COVID-19 patients should be kept in isolation
10.	Patients should disclose their exposure
11/	Medical staffs are ready to participate in anti-epidemic in the community

Attitude: Favorable 251(59.5%)

Unfavorable 171(40.5%)

Mean of attitude (33.9 ± 11.6 SD) with a range of 12 to 55

### Infection prevention practice towards COVID– 19

The prevalence of poor practice was 48.8% (95% CI (40.1–57.6%). The majority (90.8%) of the participants reuse a mask. Only one-third (33.4%) of the study participants wore a mask when leaving home to prevent COVID-19 infection. Avoiding participates in meetings, religious activities, events, and other social gatherings and frequently washing hands with soap and water for at least 20 s or using sanitizers were (37.7%), and (64.5%) respectively. The other less frequently practiced preventive measures were did not use other workers’ phones, desks, offices, or other work tools (20.4%), and “physical distancing” by the remaining 6 ft/2 m away from others (28.4%) (Table 5).

**Table 5:** Infection prevention practice of chronic disease patients toward COVID–19, Aksum Hospital, Northern Ethiopia, 2020 (*N* = 422)

Serial Number	Questions	Frequency	
		Yes N (%)	No N (%)
1.	Do you participate in meetings, religious activities, events, and other social gatherings or any crowded place in areas with ongoing community transmission?	263(62.3)	**159(37.7)**
2.	In recent days, have you worn a mask when leaving home?	**141(33.4)**	281(66.6)
3.	If yes, do you touch the front of the mask when taking it off?	121(85.8)	**20(14.2)**
4.	Do you reuse a mask?	128(90.8)	**13(9.2)**
5.	Do you wash your hands with soap and water frequently for at least 20s or use sanitizer/60% alcohol?	**272(64.5)**	150(35.5)
6.	Do you touch your eyes, nose, and mouth frequently with unwashed hands?	123(29.1)	**299(70.9)**
7.	Do you clean and disinfect frequently touched objects and surfaces?	**242(57.3)**	180(42.7)
8.	Do you practice “physical distancing” by remaining 6 ft/2 m away from others at all times?	**120(28.4)**	302(71.6)
9.	Do you use other workers’ phones, desks, offices, or other work tools and equipment?	336(79.6)	**86(20.4)**
10.	Do you limit contact (such as handshakes)?	**312(73.9)**	110(26.1)
11.	Do you eat or drink in bars and restaurants?	270(64)	**152(36)**
12.	Do you cover your nose and mouth during coughing or sneezing with the elbow or a tissue, and then throw the tissue in the trash?	**299(70.9)**	123(29.1)
13.	Do you prefer to stay at home, in a room with the window open during the transmission period?	**133(31.5)**	289(68.5)
14.	Do you stay home when you were sick due to common cold-like infection during the transmission period?	**294(72.8)**	110(27.2)
15.	Do you listen and follow the direction of your state and local authorities?	**273(67.6)**	131(32.4)

### Associated factors of poor practice towards COVID-19

Crude associations of socio-demographic variables with poor practice were checked and age, educational status, and residence were statistically significant at *p* < 0.25. These variables having significant crude associations were entered into the multivariable logistic regression model. Accordingly, rural residence, and educational status of “can’t read and write” were significantly associated with poor practice at *p* < 0.05. The odds of poor practice in rural residents of study participants were three times (AOR = 3.12, 95% CI: (2.568, 11.532)) more likely to be associated than with urban residents. This study also showed that participants who “can’t read and write” had 1.4 times (AOR = 1.4, 95% CI: (1.332, 9.612)) more likely associated to poor practice than with an educational status of “secondary and above” (Table 6).

**Table 6:** Factors associated with poor practice among chronic disease patients in bivariable and multivariable logistic regression analyses, Aksum Hospital, Northern Ethiopia, 2020 (*N* = 422)

Variable	Category	Poor Practice		OR (95% CI)		
		Yes (***n*** = 206)	No (***n*** = 216)	COR	P-value	AOR
**Age**	18–34 years	30(41.1)	43(58.9)	1		1
	35–49 years	60(36.6)	104(63.4)	1.209(1.021, 3.041)*	0.061	1.03(0.781, 5.652)
	50–92 years	116(62.7)	69(37.3)	0.415(0.321,1.209)*	0.058	1.8(0.921, 3.432)
**Sex**	Male	90(44.6)	112(55.4)	1		
	Female	116(52.7)	104(47.3)	0.72(0.421,2.31)		
**Ethnicity**	Amhara	1(16.7)	5(83.3)	1		
	Tigray	205(49.3)	211(50.7)	0.21(0.053, 3.351)		
**Residence**	Urban	130(39.2)	202(60.8)	1		1
	Rural	76(84.4)	14(15.6)	0.12(0.023,4.071)*	**0.041**	3.12(2.568,11.532)**
**Educational level**	Un able to write and read	110(60.4)	72(39.6)	2.1(1.361, 11.323)*	**0.02**	1.4(1.332,9.612)**
	Able to write and read	48(36.1)	85(63.9)	0.79(0.459,6.341)*	0.09	0.7(0.045,4.325)
	Primary school	23(48.9)	24(51.1)	1,3(1.086,7.421)*	0.12	0.76(0.576,3971)
	Secondary and above	25(41.7)	35(58.3)	1		1
**Occupational status**	House wife	42(53.8)	36(46.2)	1		
	Private business	31(34.4)	59(65.6)	2.2(2.032,5.642)		
	Government employee	16(29.6)	38(70.4)	2.7(2152,4.403)		
	Farmer	65(68.4)	30(31.6)	0.54(0.262,9.421)		
	Unemployed	38(61.3)	24(38.7)	0.74(0.432,6.592)		
	Daily laborer	5(50)	5(50)	1.2(0.943,6.347)		
	Retired	9(27.3)	24(72.7)	3.1(1.702,8.059)		

Notes: *p-value < 0.25, statistically significant in COR; ** *p*-value < 0.05, statistically significant in AOR. *Abbreviations*: *AOR* adjusted odds ratio, *CI* confidence interval, *COR* crude odds ratio. Age category [15]

## Discussion

Presently, COVID-19 has spread rapidly throughout different countries and has become a major global health concern. Consequently, understanding the ‘high-risk groups’ especially those with chronic disease, KAP and possible risk factors is necessary to prevent and control the pandemic disease of COVID-19.

According to this study, the prevalence of poor knowledge was (35.1%). This finding is higher than that of the study done in Iran (20.4%) [16] and China (10%) [17], which stated a low prevalence of poor knowledge. This might be due to a difference in the socio-economic status of study participants (in rural Ethiopia had no access to internet and electricity [18] and this results limited access of updated information related to COVID-19), differences in assessment tool used for knowledge and time of data collection.

According to this study, older age was found to be associated with poor knowledge. This is in line with a study done in Chicago [19] and Hubei Province [17]. This might be physiologically happened during aging like decrease hearing and visual ability. So, thus makes confront to read different health related issues and results poor knowledge.

This study showed that rural residents had seven times more likely associated to poor knowledge than urban residents. This finding is consistent with a study in China [17]. This might be due to there is no electricity and internet in rural areas and had lack of access to information, results poor knowledge. In addition, rural residents in Ethiopia are largely uneducated and reduced ability to comprehend health information related to prevention of COVID-19.

This study reported that participants who “can’t read and write” had 8.5 and 1.4 times more likely associated to poor knowledge and poor practice than with an educational status of “secondary and above” respectively. This finding is in line with a study in Bangladeshis [20]. This might be due to when “someone gets more educated he/she will have a better understanding of control measures and preventive strategies related to COVID-19”, and the capacity to practice recommendations to protect COVID-19 will rise. In addition, those who could read and write had an opportunity to get information through reading.

Upon this study, the prevalence of favorable attitude was (59.5%). This finding is in line with a study in China and Iran [13, 21]. This might be due to the same characteristics of the infection since this is respiratory distress disease.

In this study showed that about 71.6% of study participants strongly disagree with the statement “Ethiopia can win the battle against COVID-19 virus”. This finding is higher than that of the study in China [17] which was around 5.3% strongly disagree with the statement of “China can win the battle against COVID-19 virus”. This might be due to low traffic limits all throughout states of Ethiopia, and low socioeconomic status which decrease people’s confidence in winning the battle against the COVID-19 virus.

According to this study, the prevalence of poor practice was high (48.8%). This finding is higher than that of the study done in china [17]. This might be due to vary in sources of information, media exposure, knowledge, religious perspective, and application of governmental state of emergency.

According to this finding, rural residents had three times more likely associated to poor practice than urban residents. This is similar with a study done in China [17]. This might be due to the ways to access information in rural areas of Ethiopia are through family, friends and health care workers. When the health information is not delivered timely and resourcefully, the tendency of practicing such information is decreased.

## Conclusions

The prevalence of poor knowledge and poor practice among chronic disease patients were high. Rural residence and educational status with “cannot read and write” were significantly associated with poor knowledge and poor practice. Older age was significantly associated with poor knowledge. The health care workers at the chronic outpatient department should provide detailed information about COVID-19 to their patients. The patients should get information about COVID-19 in their local languages.

## Data Availability

The datasets used and/or analyses during the current study are presented within the manuscript and available from the corresponding author on reasonable request.

## Abbreviations

ARDS: Acute Respiratory Distress Syndrome
COVID–19: CoronaVirus Disease in 2019
KAP: Knowledge Attitude and Practice
MSC: Masters of Science
SARS: Severe Acute Respiratory Syndrome
SD: Standard Deviation
SPSS: Statistical Package for the Social Science
WHO: World Health Organization
